# Is the pre-natal period a missed opportunity for communicating with parents about immunizations? Evidence from a longitudinal qualitative study in Victoria, British Columbia

**DOI:** 10.1186/s12889-022-12658-3

**Published:** 2022-02-05

**Authors:** Clara Rubincam, Devon Greyson, Constance Haselden, Robin Saunders, Julie A. Bettinger

**Affiliations:** 1 Island Health, Victoria, BC Canada; 2 Vaccine Evaluation Center, BC Children’s Hospital Research Institute, University of British Columbia, A5-950 West 28th Street, Vancouver, BC V5Z 4H4 Canada; 3 Department of Communication, University of Massachusetts, Amherst, USA; 4 University of British Columbia, Vancouver, BC Canada; 5 South Island Division of Family Practice, Victoria, BC Canada

**Keywords:** Vaccine, Vaccination, Parent, Decision-making, Interviews, Health care provider, Midwives, Doctors

## Abstract

**Background:**

Growing evidence shows that many parents begin the decision-making process about infant vaccination during pregnancy and these decisions – once established – may be resistant to change. Despite this, many interventions targeting vaccination are focused on communicating with parents after their baby is born. This suggests that the prenatal period may constitute a missed opportunity for communicating with expectant parents about infant vaccination.

**Methods:**

Using a longitudinal qualitative design, we conducted two interviews (prepartum and postpartum) with women (*n* = 19) to explore the optimal timing of vaccination information. The data were analyzed thematically, and examined across all sets of pre- and post-partum interviews as well as within each individual participant to draw out salient themes.

**Results:**

Most participants formed their intentions to vaccinate before the baby was born and indicated that they would welcome information about vaccination from their maternity care providers. However, few individuals recalled their maternity care providers initiating vaccination-related conversations with them.

**Conclusion:**

The prenatal period is an important time to begin conversations with expectant parents about vaccinating their infants, particularly if these conversations are initiated by trusted maternity care providers. More information is needed on how maternity care providers can be better supported to have these conversations with their patients.

## Background

Vaccines are an effective public health intervention to combat a variety of communicable diseases [1], yet pediatric immunization rates remain suboptimal in Canada [2], especially in the province of British Columbia (BC) [3–6]. These low immunization rates are often attributed to parental distrust or suspicion about the safety of vaccines [7]. Beyond the estimated 12% of parents who refuse all or some of the recommended vaccines, a recent Canada-wide survey indicated that even among parents who accepted all recommended vaccines, 40% reported “doubts and concerns with vaccinating my child” [6]. Moreover, respondents from British Columbia were more likely to refuse all vaccines compared to the national average [6].

Growing evidence shows that many women begin the decision-making process about infant vaccination during pregnancy [6, 8–13] and these decisions – once established – may be resistant to change [14]. Existing studies have documented key concerns about vaccination among pregnant women [15, 16] and new parents [17–19] that required skilled communication and thoughtfully disseminated information from trusted health care sources. Despite the evidence on the importance of the pre-natal period as a decision-making time, interventions to address vaccination concerns largely center on doctor-parent communication post-childbirth [20–22]. As a result, the pre-natal period may be an underutilized opportunity for initiating communication about vaccinations [23]. Few studies, to date, have examined the evolution of beliefs and practices about vaccination during pregnancy and through the child’s first months of life [14, 24, 25]. To our knowledge, none have been conducted in British Columbia.

To explore the potential for vaccination communication prenatally, this study examined women’s decision-making processes about pediatric vaccinations during two distinct time periods: the third trimester of pregnancy and 4-6 months after birth, in order to explore the optimal time to provide parents with information about infant vaccines, and identify mothers’ perceptions of the ideal source of information about immunizations.

## Methods

This longitudinal qualitative study [26, 27] recruited English-speaking participants through maternity care providers (physicians and midwives), complementary and alternative health care providers (acupuncturists and naturopaths), and pre-natal classes offered through a regional health authority, a local college, and a parenting resource center in Victoria, British Columbia, Canada. Women choosing midwifery care as well as those under physician care were deliberately sought, as some studies have found that mothers with midwifery-assisted birth are more likely to delay vaccinations, vaccinate selectively, or not vaccinate at all [19, 24, 28]. The model of maternity care differs for each patient in British Columbia, but in general, a woman receives maternity care from either a midwife, a specialist obstetrician/gynecologist, or a family physician with a maternity practice. This may be in addition to a relationship with a regular family physician, though an increasing number of patients are “unattached”, meaning they do not have a regular family physician to provide longitudinal care outside of maternity care. In British Columbia, longitudinal health care providers (family doctors) typically provide care to infants and young children, and therefore could be the providers of vaccinations.

An exploratory and descriptive model was used for this study as the main purpose was to better understand the decision-making processes of expectant and new mothers [29, 30]. Purposive sampling was used to ensure diverse views on child vaccination intentions and beliefs by recruiting pregnant women and using a preliminary screening question,“What would you say is your overall perspective on children’s vaccinations?” [31]. Participants were not screened for ethnocultural or income diversity. A related arm of this research project involved speaking with some of the co-parents of participants; these data indicated the vast majority of vaccination decisions were made by the mothers (as all partnerships were male-female). As a result, the data from fathers were not considered to be salient to the current analysis and were excluded from this in-depth exploration of mothers’ decision-making processes.

### Data collection

The first round of individual interviews began in October 2015 and the final interviews of the second round were completed in November 2016. Both the first and second interviews were semi-structured and iterative in nature, which enabled the interview guide to be adjusted in response to emerging lines of inquiry throughout data collection. Interviews ranged in length from 30 to 90 min and were audio-recorded. The first interviews, completed during the third trimester of pregnancy, sought to establish mothers’ beliefs and intentions regarding vaccination. All first interviews were conducted in person, either at the interviewee’s home or in the offices of the local public health unit or the prenatal and postpartum resource center from where they were recruited. The second interviews occurred 4-6 months after birth and followed up on mothers’ beliefs and practices regarding vaccination, with particular attention to how these may have evolved or solidified since the first interview [10], whether mothers had initiated 2- and 4-month vaccinations, and their intentions towards subsequent vaccinations.^1^ The second interview was either in person at the interviewee’s home or over the phone, depending on interviewee preference. The interviewer was one of the co-authors of the study, a university-affiliated researcher. For a portion of the interviews, the interviewer was pregnant, a fact known to participants. The study was approved by the University of British Columbia Children’s and Women’s Health Centre of British Columbia Research Ethics Board (H15-01709). Participants provided written informed consent, and received a $25 gift card for a local grocery store as compensation for their time after each interview.

Recruitment, data collection, and data analysis for the first round of interviews were conducted until data saturation was achieved, defined as when no new themes emerged after 3 successive interviews [32–34]. We mitigated attrition between study waves by conducting most interviews on site (e.g. at expectant and new mothers’ homes) or at the local pregnancy and birth resource center, and no participants were lost to follow up.

### Analysis

During analysis and write-up, participants were assigned letter codes to further protect their identities and privacy, and all identifying information was redacted from transcripts. Transcripts were inductively coded using NVivo 11 (released in 2015) by one author into main themes and sub-themes. This thematic analysis assigned preliminary codes to the data to describe the content (for example: ‘trust in maternity care provider’) [30, 35]. This was followed by identifying and discussing patterns of themes with the larger study team [36]. Initial analysis proceeded cross-sectionally, enabling exploration of emerging themes as the prenatal interviews were being completed. Once both interviews were complete, analysis was conducted longitudinally, by reading the first and second interview transcripts for each participant together as a single case. This enabled exploration of how each mother described the evolution of her vaccination decision making over time, and ultimately yielded the dominant themes of the optimal *timing* of and *source* of information about infant vaccinations, which are described in this paper.

## Results

A total of 19 English-speaking mothers participated in the first and second round of interviews, with no attrition between interview waves. Participants reported a variety of socio-economic backgrounds and ages (see Table 1), although those with higher educational outcomes and higher household incomes were disproportionately represented. The majority (84%) were first-time mothers, in part due to the fact that multiparous women may have less time for research. Eleven participants (58%) were in the care of midwives. This represents an oversampling of this population as approximately 20% of pregnant women in BC are in the care of midwives, and 15% of women have a midwifery-attended birth. Overall, the sample disproportionately represents English-speaking first-time mothers with higher household incomes, higher educational attainment, and under midwifery care.

**Table 1:** Characteristics of Study Participants (*N* = 19)

	n (%)
**Household Income**	
Less than $50,000	4 (21.05%)
Between $50,000-$100,000	7 (36.84%)
More than $100,000	7 (36.84%)
Opted to skip the question	1 (5.26%)
**Education**	
Certificate/Diploma	4 (21.05%)
Bachelors Degree	11 (57.5%)
Masters Degree	3 (15.79%)
PhD or above	1 (5.26%)
**Marital Status**	
Married	14 (73.68%)
Common law	5 (26.32%)
**Employment Status**	
Employed full time	15 (78.95%)
Unemployed	3 (15.79%)
Homemaker	1 (5.26%)
**Maternity care provider**	
Midwife	11 (57.5%)
Family Doctor (providing both primary and maternity care)	1 (5.26%)
Family Doctor (providing maternity care only)	5 (26.32%)
Obstetrician/Gynecologist	2 (10.53%)
**Number of children**	
First time mother	16 (84.21%)
Has previous children	3 (15.79%)
**Longitudinal care**	
Yes – has regular family doctor	16 (84.21%)
No – does not have regular family doctor	3 (15.79%)
**Age**	
Mean	33 (range 24-38)

Through these interviews, certain common themes emerged about when participants formed their intentions to vaccinate, who they trusted to inform this decision, and when they would have preferred to receive information about vaccination. The majority of participants formed their intentions to vaccinate during the prenatal phase, although few recalled receiving formal guidance from their health care provider (HCP) about this decision. They described the quality of their relationships with their prenatal and postnatal care providers in terms of the level of trust they placed in the health information they provided. Participants’ narratives indicated the prenatal period was the optimal time for vaccination information delivery, and that prenatal care providers were the optimal sources of information about vaccination.

### Prenatal intentions to vaccinate

At the time of the first interview (third trimester of pregnancy), most participants (*n* = 15) had formed their vaccination intentions. No noticeable differences were observed in this regard between primiparous and multiparous participants. Asked about her intentions, one second time mother stated, “Oh we’re definitely going to vaccinate. I vaccinated my daughter” (Participant L, Pre-natal interview^2^). Another participant explained, “We will be vaccinating?... Probably the full schedule”. (PQ, Pre). A first-time mother stated her comfort with following the official guidelines, saying, “I’m happy to go with just what is the standard practice” (PF, Pre).

Four participants – all first-time mothers who had reported higher levels of doubt or concern around vaccines—had not formed a clear intention regarding vaccination at the time of the prenatal interview. These women spoke about the need for more information, to do their own research, to speak with a trusted health care professional, and to give the decision about vaccination more thought. One first time mother explained:

I think, I mean I think it’s something that we’ll look into, but again, I don’t know much about it for babies, and I don’t know the timing and when anything actually happens, so it’s probably just something that we’ll have to talk about more. Learn more about with the doctor and stuff when we get there [PB, Pre).

Referring to a family member who had previously had an unexpected reaction following vaccination, one participant responded to the question about intentions regarding vaccination:

P: Um, if I had to say right now I’d say probably selectively.

I: Mhmm.

P: Um, maybe delayed if our son had a poor reaction like my brother did

(PO, Pre).

Another refused to answer definitively, saying “I’m not claiming that we’re not going to vaccinate and I’m not claiming that we are” (PI, Pre).

One participant was reserving her final decision about whether to administer the full infant vaccination schedule or a selective approach until she had confirmation from her family doctor:

P: I don’t know enough.

I: Yeah. So you’re sort of waiting for the recommendation from your doctor to make that decision?

P: Yeah (PB, Pre).

In review, the dominant theme in this population was to have already decided to follow the vaccine schedule while participants were still pregnant. Notably those who were undecided on whether or not to follow the schedule indicated they were awaiting a further conversation with their health care providers, in the hopes to clarifying some areas of concern including side-effects and adverse reactions.

### Conversations with maternity care providers about vaccination

Although most participants had established intentions regarding vaccination by the final trimester of the prenatal period, few recalled their maternity care providers initiating any substantial communication about infant vaccination, although they did recall conversations about other newborn interventions such as vitamin K injection and vitamin D supplementation. This indicates that many participants felt they had formed their intentions independently of the advice or consultation of their maternity health care professional. We did not observe any differences in this regard between first time mothers and mothers with other children, or among participants whose maternity care providers were midwives, family physicians, or obstetricians.

Participants under the care of midwives were almost unanimous when asked whether they had spoken with them about vaccination during the prenatal phase:

P: Ummmmm, not really. I don’t [pause] think that’s in their scope of practice (PF, Pre, with a midwife providing maternity care).

P: She (midwife) said that she’s not really qualified to offer any – like, they’re not really taught a lot about vaccinations – they’re taught to – you know – the specific ones that might be of interest at certain points in the pregnancy or in the six weeks postnatal or whatever, but yeah, she said I’ll try to find you some stuff online and then the next time I saw her she said ‘Well, there’s this one website but it seemed kind of angry’. And I said, ‘Yeah, I’ve probably found that one’. (PI, Pre, in midwifery care).

Asked at the follow-up interview to recall whether her health care provider discussed vaccinations during the pregnancy, this same participant confirmed:

P: She did not, not at all. I asked her about them and she was like “I’m sorry I can’t advise you, I don’t know anything” (PI, Post, in midwifery care).

One participant thought these discussions were coming in the postnatal phase, stating, “No they haven’t [initiated this conversation yet]. I suspect she probably will maybe after the birth.” However, after this participant’s delivery, she described being referred to the public health unit rather than having a conversation with her midwife, “I think she just said, you know, your vaccinations are being done at the public health unit. I think is pretty much what she said.” (PQ, Post).

Although the infant vaccination schedule was not a topic of discussion, participants reported that their maternity care providers spoke with them about other types of early health interventions for their infants:

I: Your midwives haven’t talked to you about vaccination after the baby’s born?

P: No.

I: Did they talk to you about Vitamin K?

P: Yes, um hum (PB, Pre).

When asked if her midwives had raised the topic of infant vaccination yet, one woman responded,

Not actually for the baby yet except they said--it is Vitamin K shot right at the beginning--so that’s not really vaccinations. Um, so we haven’t really discussed those immediate things, which maybe we should have (PJ, Pre).

Participants reported the absence of discussions about vaccination across multiple sources of prenatal education:

I: Has your maternity doctor talked about vaccines at all with you?

P: Uh no.

I: And then, the prenatal class here, did they talk about vaccines at all?

P: No, it was like labour and delivery class and stuff like that, and like breastfeeding stuff (PC, Pre).

Those participants with a primary care family doctor also reported a lack of conversations about vaccination in the prenatal phase. One woman had seen her family doctor for her own health issues during the pregnancy and they had discussed seeing each other after the delivery: “Um, but he didn’t mention, you know, ‘and then we’ll talk about the vaccination schedule’. He didn’t really say any of that” (PF, Pre). This same participant’s midwives did not engage in-depth with the topic of vaccinations either:

I: Did your midwives talk to you about vaccination? Either in the – after I saw you in the prenatal phase, or in the post partum visits?

P: Um, I don’t think so. I believe they may have said, like, you should phone them [the public health units] soon to schedule, or something like that…Because they’re kind of behind [overbooked], but I don’t think we really had a conversation about vaccinations (PF, Post).

Another participant with a primary care family doctor echoed this sentiment, saying “I don’t think it’s something that’s really come up”. (PG, Post). Some maternity care providers may have avoided the topic with mothers they felt were already decided about vaccination. When asked whether her maternity doctor had spoken with her about infant vaccines, one first time mother reported “No, ‘cause he knew that I wanted to give my daughter shots and make sure that she gets them” (PC, Post).

A few participants took a more proactive approach to initiating these conversations with their maternity care providers about immunizations. One participant shared that “we wanted to know what he [maternity doctor] would do with his own kids” (PD, Post), and having received a satisfactory answer, proceeded with vaccination herself. However, not all participants reported getting satisfactory answers to their questions:

I think I asked a little bit – I actually asked her why Hepatitis B was included [in the infant schedule] and she said she didn’t know. She said her kids were not vaccinated for it, but they were born in Ontario when they were younger and they didn’t do it out there…But yeah, I don’t think we really had a -- my midwife and I did not have a formal discussion about it (PB, Post).

In short, though few women reported any discussions about infant vaccines during the prenatal period with their maternity care providers, many wanted such discussions to occur.

### Women’s trust in their maternity care providers

For participants who had a regular family doctor before they became pregnant (*n* = 16), the majority of them were receiving maternity care from a different HCP than the one who provided their longitudinal primary care. As well, a subset of participants (*n* = 3) did not have a regular family physician before or after their pregnancy. As a result, most participants in this study received their maternity care from someone other than their regular family physician, either because they chose a different model of care (i.e. midwife), or because they did not have a regular family doctor. Participants in this study described the quality of their relationships with their maternity and primary care providers in contrasting ways. Most participants reported a relatively strong, trusting relationship with their maternity care providers, regardless of provider type. One woman, who had chosen a midwifery practice for her care, described the quality of care:

I never felt rushed. They were always happy to answer my questions especially in the first trimester when I was vomiting constantly. I was really worried and they were really responsive to emails and—things like that and not that the doctor route necessarily would have been that different, I don’t know, but I like the extent of the care I was getting (PM, Pre).

Another participant described the aspects of maternity care that were important to her:

P: She’s [midwife] very relaxed. She’s very gentle. She’s excellent at explaining her motivations behind decisions.

I: Mmm.

P: She’s really excellent at making sure that I have all the information I need, and she’s certainly not afraid to bring in other experts when she feels that there’s a need. So yeah, she’s very informed and just I think a really excellent practitioner.

(PQ, Pre).

A participant with an OBGYN expressed confidence in their care of her uncomplicated pregnancy:

I: You feel good about the care, and you feel confident going forward –

P: I feel like if something was wrong, he would probably would spend more time with me but there’s no reason to, which is fine (PC, Pre).

A patient with a maternity doctor felt confidence in the quality of her care:

I feel like she’s really supportive of me and she only does maternity patients so I feel like it’s really focused care (PG, Pre).

As a way of illustrating her trust in her provider’s recommendation, one woman explained her plans for the influenza vaccine for herself:

P: Well, I’m going to go see my midwife today so I was going to actually ask her today about it [influenza vaccine] [laughter].

I: Okay, so you’ll sort of see whether she recommends it?

P: Yeah.

I: Do you feel like that would change your decision around it if she--?

P: Yeah, yeah, it probably would ‘cause I trust what she has to say.

I: Mmm, okay, so if she came down strongly in favour that you would do it?

P: Yeah (PL, Pre).

In contrast to the close, trusting relationships described by participants between themselves and their maternity providers, the majority of women did not report a longstanding or overly trusting relationship with their longitudinal HCP. Some women in the sample had only recently secured a spot in a family practice, others had a regular HCP that they were ambivalent about, while others did not have a regular family doctor and remained largely dependent on the walk-in clinic system for their longitudinal health care needs.

Participants described their relationship with their longitudinal HCP in the following terms:

P: I have a family doctor. Um…its not anyone that I feel close to at all. I don’t even think she knows I’m pregnant. Yeah. So. [Laughs] (PE, Pre).

P: Uh, my previous family doctor just retired so I don’t really know much about him, but I do have a family doctor (PJ, Pre).

A common theme was of participants feeling fortunate to have secured a primary care family physician, regardless of the quality of the relationship. One woman stated, “Yes, I’m extremely excited that I actually even have a family doctor to see. ‘Cause I realize that they can’t turn down pregnant women but when you’re not pregnant anymore, they, like, if you’re just going to see a family doctor, I don’t think they have to keep you around” (PC, Pre).

Another participant echoed this sentiment of feeling privileged to have access to longitudinal primary care, saying:

P: My family doctor is – she’s in town though, kind of in this area. So it’s a bit of a drive. I’m debating whether or not to stay with her or to switch – to try to switch to somebody closer but it’s so hard to get into [a practice]. So I haven’t gone about putting my name on wait lists yet” (PM, Pre).

Some participants were unattached – without a longitudinal HCP - in the first interview, and a few remained so in the second. When asked who was looking after her and her baby in the postpartum phase, one woman responded simply, “No family doctor. They are hard to come by” (PO, Pre).

This lack of longitudinal primary care caused some participants to wonder about where to go for more information about their baby’s health. One participant without a primary care provider commented:

The more I think about it, the more I realize how much I don’t know. And I don’t actually know where to find that information. And I mean, when you have a child, I’m sure people care a lot more. But even as an adult, I don’t know - there’s a lot I don’t know about vaccinations, so, it will be interesting to, I guess, I don’t know, find some resources for that (PH, Pre).

Particularly in contrast to their relationships with their primary care providers, participants’ close and trusting relationships with their maternity care providers underscores the importance of using this time to initiate and follow-up on conversations about vaccinations with expectant mothers.

## Discussion

The majority of participants in this study reported having formed an intention about infant vaccinations by the time their baby was born. Most planned on vaccinating their infant according to the provincial schedule and without delays. Regardless of whether they were planning to vaccinate or not, some participants described persistent uncertainty or concerns about vaccines in the prenatal phase. They were readily able to articulate their questions or concerns during this phase, and appeared eager to receive advice and additional information from trusted health care providers.

Most participants described their maternity care providers as competent, trustworthy, and generally capable of explaining health issues to them in ways that brought deeper confidence and understanding. However, despite suggesting that they would be receptive to their maternity care providers’ recommendations about infant vaccinations, few women reported that these providers had initiated conversations about infant vaccinations, either in the prenatal phase or the postpartum period. These findings reinforce and add context to other studies indicating that the overwhelming majority of women wish to receive vaccination information well in advance of the vaccination appointment. Wu et al. found that 70% of participants wanted information about vaccines during pregnancy, although only 18% reported receiving this information during the prenatal period [37]. Similarly, Vannice et al. reported that more than 95% of participants expressed a preference for receiving vaccination information during pregnancy or prior to the vaccination appointment [11]. In our study, when participants asked their provider specifically about infant vaccines, their maternity care providers deferred to other HCPs or suggested the participant seek answers elsewhere.

The lack of prenatal communication about the infant immunization schedule may not be of significant concern for those women who remain confident about their infant immunization decision throughout the pregnancy and postpartum period. However, for those women who experience doubts about infant vaccination in the prenatal phase, the absence of discussions with their maternity care provider constitutes a missed opportunity to elicit information and recommendations from a trusted professional. These parents may be ‘hesitant compliers’, as Enkel and colleagues describe, those who fully vaccinate but still report concerns [38], or they may be those who selectively vaccinate, delay vaccination or refuse vaccinations altogether. Glanz et al. (2013) found that parents with doubts or concerns were more likely to begin the deliberation process about infant vaccinations earlier than parents who unquestioningly accepted vaccines and recommended engaging obstetricians to address these concerns [10]. In a recent national survey of Canadian parents, those reporting low trust in vaccines were more likely to say the vaccination decision was difficult [6]. Harmsen et al’s (2013) study suggested parents who refused all or some vaccines did not feel they were receiving sufficient information from official sources [39].

These findings build on data from earlier studies suggesting that parents who are hesitant towards vaccination are particularly receptive to counsel about vaccination from sources they perceive to be ‘alternative’ to traditional or allopathic medicine, including midwives [18, 24, 39]. It further supports existing findings that the prenatal period is an invaluable window of opportunity to disseminate vaccination information to hesitant parents at a time when they are information seeking and open to trusted sources of information [10–14, 40–46]. Other studies suggest that the postpartum period is not an optimal time to receive information about vaccines because parents do not feel they have adequate time to assess new information due to the exhaustion of dealing with a new baby [14, 40–42, 47, 48]. A recent systematic review of parents’ views on vaccination communication found that parents preferred information to be communicated well in advance of their date of vaccine administration [49] to provide adequate time for reflection and decision-making.

While these findings highlight the importance of early communication from a health care provider, it is important to note that not all clinician-patient relationships are predicated on high levels of trust. While many interventions to promote vaccination are based on a “strong physician-parent relationship” [22, 50–52], the findings from this study serve as a reminder that some patients view their relationships with their longitudinal HCP as functional or adequate at best, and few reported a long-standing, trusting relationship with this provider. Participants’ trust in their maternity care providers takes on increased significance in the context of changes in the provision of primary care and public health services across Canada. Statistics Canada suggests that the rate of unattached patients (i.e. no family physician) is on the rise [53], meaning that increasing numbers of Canadians will be without a regular family physician. These changes intensify the importance of the trusting relationship women describe with their prenatal care providers, particularly for those parents with doubts or concerns about vaccines. While those who are hesitant are still deliberating about the vaccination decision, these maternity care providers may constitute the best opportunity for them to elicit the advice of a trusted health care professional [24].

Further research is needed to explore the extent to which Canadian maternity care providers feel that discussions about infant immunizations are within their scope of practice, in line with studies from other countries [54–57]. Recent research by some study authors with BC midwives indicate that while the majority of BC midwives do discuss vaccines with their clients at some point in their care, those who do not often cite a desire to avoid pressuring patients or make them uncomfortable [58].

Few studies to date have tested the effect of informational interventions delivered during the prenatal phase on postnatal vaccination uptake rates. One study in Japan found that intentions to vaccinate and vaccination uptake were higher among parents who received prenatal education about vaccinations [59]. A US study found vaccination knowledge was higher among those who received a prenatal education intervention, although no differences were noted with regards to infant vaccine initiation [60]. Another study found no difference in positive views towards vaccination among mothers who received vaccine information materials before the 2-month postpartum visit and those who received it during the 2-month visit. However, 95% of these study participants still stated they wished for vaccine education materials to be provided to them during pregnancy or prior to the 2-month vaccination visit [11]. More research is needed to explore the impact of earlier initiation of vaccination information delivery on vaccination uptake. It may be the earlier timing of vaccination information is a necessary but not sufficient element in the vaccination decision, with the source of vaccination information being of equal or greater importance. Given the close and trusting relationships with maternity care providers reported by women in our study, a vaccine recommendation followed by additional vaccination information from the maternity care provider may be important. What emerges from our study, and reinforces earlier work, is a clear consensus that women wish for opportunities to review infant/child vaccination information during pregnancy. Taken together, these findings and data from our study suggest vaccination information should be provided in an ongoing and sustained manner, beginning in pregnancy and ideally continuing on throughout the infant’s first months of life.

## Limitations

This study was a longitudinal qualitative study and thus results cannot be generalized beyond the study population or translated into quantifiable results. Though every effort was made to recruit participants from a wide variety of health care provider types, locations in Victoria, and socio-economic circumstances, participants with lower household incomes, those with less educational attainment, and those experiencing employment or housing instability were underrepresented or not represented as participants in the study. First time mothers were also more likely to participate, and it may be that the additional time commitment for interviews made it challenging for multiparous women to participate in this study. It is also possible the identity of the researcher influenced the nature of the findings, as participants shared insights from their pregnancies and post-partum life with another woman who was visibly pregnant. Findings from the co-parents (all fathers in this sample) were not reported alongside these data, as the majority of mothers indicated they were the sole or primary decision-maker with regards to their child’s vaccination. However, this could be explored in future studies with a more socio-economically, linguistically, and ethnoculturally diverse sample, as these may reveal important variations in decision-making around health care. We would caution against any effort to generalize these findings beyond the study population. However, the general themes identified by participants resonated closely with findings from other studies and highlight the importance of pregnancy as a crucial time of information gathering and decision making regarding infant vaccination.

## Conclusion

This study provides detailed evidence of the importance of the pre-natal period in the decision-making process about infant vaccination, and highlights the significant role maternity care providers can play in discussions with pregnant clients about infant vaccines. Further research on current maternity care providers’ practices around recommending infant vaccination may help inform efforts to enlist this trusted body of professionals in the crucial task of advocating for, and educating about infant vaccines.

## Data Availability

The datasets generated during the current study are not publicly available due to the fact that permission was not sought at the time of participant interviews to share recordings or transcripts outside of the research team.

## Abbreviation

HCP: Health care provider

## Footnotes

1 Each province in Canada determines their own vaccination schedule. The schedule in British Columbia involves vaccines at 2- (DtaP-HB-IPV-Hib, Men-C, PCV13, and RotaTeq), 4- (DtaP-HB-IPV-Hib, PCV13, and RotaTeq), 6 months (DtaP-HB-IPV-Hib, and RotaTeq).

2 For the duration of the article, the participant code will be shortened to ‘PL’, ‘PQ’, etc. to refer to the participant letter, and ‘Pre’ or ‘Post’ to refer to the timing of the interview.
